# Mechanisms of Action of Mesenchymal Stem Cells in Metabolic-Associated Fatty Liver Disease

**DOI:** 10.1155/2023/3919002

**Published:** 2023-01-06

**Authors:** Sitong Yi, Qingwei Cong, Ying Zhu, Qiumin Xu

**Affiliations:** ^1^Department of Infectious Disease and Liver Disease Center of Integrated Traditional Chinese and Western Medicine, The First Affiliated Hospital of Dalian Medical University, Dalian, Liaoning, China; ^2^The First Affiliated Hospital of Dalian Medical University, Dalian, Liaoning, China

## Abstract

Metabolic-associated fatty liver disease (MAFLD) is currently the most common chronic liver disease worldwide. However, its pathophysiological mechanism is complicated, and currently, it has no FDA-approved pharmacological therapies. In recent years, mesenchymal stem cell (MSC) therapy has attracted increasing attention in the treatment of hepatic diseases. MSCs are multipotent stromal cells that originated from mesoderm mesenchyme, which have self-renewal and multipotent differentiation capability. Recent experiments and studies have found that MSCs have the latent capacity to be used for MAFLD treatment. MSCs have the potential to differentiate into hepatocytes, which could be induced into hepatocyte-like cells (HLCs) with liver-specific morphology and function under appropriate conditions to promote liver tissue regeneration. They can also reduce liver tissue injury and reverse the development of MAFLD by regulating immune response, antifibrotic activities, and lipid metabolism. Moreover, several advantages are attributed to MSC-derived exosomes (MSC-exosomes), such as targeted delivery, reliable reparability, and poor immunogenicity. After entering the target cells, MSC-exosomes help regulate cell function and signal transduction; thus, it is expected to become an emerging treatment for MAFLD. In this review, we comprehensively discussed the roles of MSCs in MAFLD, main signaling pathways of MSCs that affect MAFLD, and mechanisms of MSC-exosomes on MAFLD.

## 1. Introduction

Metabolic-associated fatty liver disease (MAFLD) is previously known as nonalcoholic fatty liver disease (NAFLD). It is estimated to affect one-fourth of the global population. The increasing incidence of overweight and obesity, diabetes, hyperlipidemia, and other metabolic diseases also led to an increase in MAFLD cases [1]. Based on a progressive understanding of the disease, a team of international experts drafted a proposal to rename NAFLD to MAFLD, defined the new diagnostic criteria, removed the amount of alcohol consumed as a necessary condition to diagnose MAFLD, and ensured that MAFLD is a single and clear entity rather than a simple “antidefinition” disease [2]. The pathophysiology of MAFLD involves various interrelated processes. Insulin resistance is the driving factor of liver steatosis in MAFLD. Lipotoxicity caused by the accumulation of various toxic lipids and inflammatory reaction caused by immune system activation are the major factors involved in nonalcoholic steatohepatitis (NASH) and liver fibrosis. Endoplasmic reticulum stress and mitochondrial dysfunction further caused by lipotoxicity eventually lead to cell death [3]. MSCs have self-renewal ability and can be separated and extracted from the umbilical cord blood, bone marrow, placenta, cartilage, urine, and many other organs and tissues [4–6]. Signals of injury generated by the body trigger the migration of MSCs to the injury site and further differentiate into regional progenitor cells to substitute aging or apoptotic cells. HLCs are cells with similar functions and characteristics to normal hepatocytes. It has been proved in vitro and in vivo that MSCs could be induced to differentiate into HLCs and express hepatocyte specific markers, such as albumin (ALB) [7, 8]. Recent studies have revealed that MSCs can alleviate hepatic injury, improve hepatic function, and promote liver tissue regeneration and repair. Various liver diseases can be treated with MSC transplantation [9–11]. At present, various animal experiments of MSC transplantation have been applied to MAFLD (Table 1). Different cytokines and growth factors released by MSCs through the paracrine pathway have immunomodulatory properties and inhibit the progression of liver inflammation and liver fibrosis [12]. MSCs can also regulate lipid metabolism and improve hepatocyte steatosis [13]. Recent experiments and studies have reported that the exosomes released by MSCs through paracrine action have demonstrated excellent results in the treatment of liver diseases. Exosomes not only have the characteristics of MSCs but can also carry proteins, lipids, microRNAs (miRNAs), long noncoding RNAs (lncRNAs), and other bioactive substances. After entering the target cells, they exert immunomodulatory function, further inhibit the development of fibrosis, and promote liver tissue regeneration [14]. Therefore, MSCs and their exosomes may play a significant function in the occurrence and progression of MAFLD.

## 2. Roles of MSCs in MAFLD

**Figure 1:**
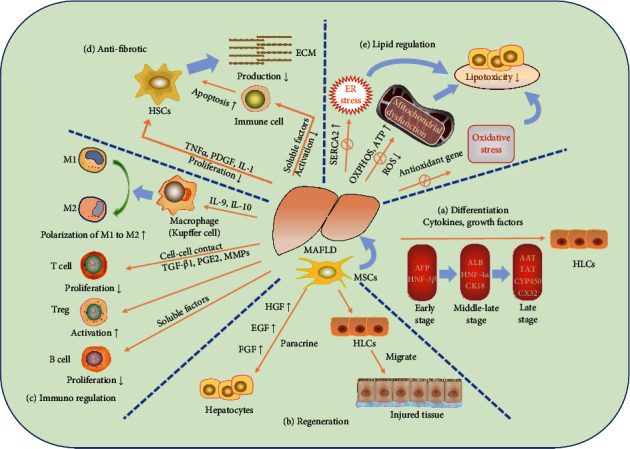
Mechanisms of MSCs in MAFLD. (a) MSCs can differentiate into HLCs under the actions of cytokines and growth factors. (b) MSCs can promote liver tissue regeneration. (c) MSCs regulate the corresponding effector cells of innate and adaptive immune response. (d) The antifibrosis effect of MSCs can be divided into direct and indirect effects on HSCs. (e) MSCs reduce lipid toxicity by regulating lipid metabolism. MSCs: mesenchymal stem cells; HLCs: hepatocyte-like cells; AFP: alpha fetoprotein; HNF-3*β*: hepatocyte nuclear factor-3*β*; ALB: albumin; HNF-4*α*: hepatocyte nuclear factor-4*α*; CK18: cytokeratin 18; AAT: *α*1-antitrypsin; TAT: tyrosine aminotransferase; CYP50: cytochrome P450; CX32: connexin 32; HGF: hepatocyte growth factor; EGF: epidermal growth factor; FGF: fibroblast growth factor; IL-9: interleukin-9; IL-10: interleukin-10; TGF-*β*1: transforming growth factor-*β*1; PGE2: prostaglandin E2; MMPs: matrix metalloproteinases; TNF-*α*: tumor necrosis factor-*α*; PDGF: platelet-derived growth factor; IL-1: interleukin-1; HSCs: hepatic stellate cells; ECM: extracellular matrix; SERCA2: sarcoplasmic reticulum Ca(2+) ATPase; ROS: reactive oxygen species.

MAFLD is considered a complex disease ranging from simple steatosis to NASH, liver fibrosis, cirrhosis, and hepatocellular carcinoma (HCC). During disease development, liver inflammation and hepatocyte injury caused by various factors cannot be ignored. Therefore, it is important to explore the potential roles of MSCs in the pathogenesis of MAFLD for the development of new therapies (Figure 1).

### 2.1. Differentiation Ability of MSCs

MSCs can self-renew and differentiate into various progenitor cells, such as hepatocyte-like cells (HLCs), with actions similar to general hepatocytes [27]. Thus, is it possible that MSCs differentiate into HLCs to replace damaged hepatocytes in MAFLD? At present, experiments have confirmed that human MSCs that separated from bone marrow [28], adipose tissue [29], umbilical cord blood [30], and other organs and tissues can induce differentiation into HLCs under certain conditions. Studies have also reported that MSCs differentiate into hepatocytes in rats [31], sheep [32], and humans [33]. Hormones and some soluble factors can affect the ability of MSCs to differentiate. For example, MSCs can differentiate into HLCs with hepatocyte-characteristic phenotypes and functions under the action of transforming growth factor (TGF), epidermal growth factor (EGF), insulin-like growth factor, and oncostatin M [34]. The differentiation of MSCs into hepatocytes include three stages: initiation, differentiation, and maturation. In the early stage of MSC differentiation into hepatocytes, cells express biomarkers such as alpha fetoprotein (AFP) and hepatocyte nuclear factor-3*β* (HNF-3*β*). Then, middle-late stage biomarkers were expressed, for example, albumin, hepatocyte nuclear factor-4*α* (HNF-4*α*), and cytokeratin 18 (CK18) [30, 35]. In the late stage, cells express biomarkers the same as mature hepatocytes, e.g., *α*1-antitrypsin, tyrosine aminotransferase, cytochrome P450, and connexin 32 [30, 35]. Among the markers, the most studied ones were plasma proteins (ALB and AFP), transcription factors (HNF-3*β* and HNF-4*α*), and cytoskeletal proteins (CK18 and CK8). Lin et al. [8] found that transplanted human MSCs differentiated into HLCs in both in vivo and in vitro studies. They transplanted human MSCs into rats with liver fibrosis through the portal vein, and human albumin positive cells were detected 21 days after transplantation, indicating that transplanted MSCs successfully differentiated into HLCs with normal liver cell function and expressed albumin, hepatocyte growth factor (HGF), and metalloproteinases in vivo. According to this finding, Winkler et al. [36] transplanted human bone marrow-derived mesenchymal stem cells (BM-MSCs) into NASH mice. Human HLCs were found in the liver parenchyma of NASH mice 7 days after transplantation, and triglycerides (TGs) were reduced in the liver, and the expression of proinflammatory cytokine tumor necrosis factor- (TNF-) *α* was decreased, reversing the liver injury of NASH mice. Li et al. [15] transplanted human umbilical cord-derived mesenchymal stem cells (UC-MSCs) into the liver of type 2 diabetes mellitus (T2DM) MAFLD mice and observed that the expressions of transaminase decreased and HNF-4*α* increased, which restored the liver function. However, the specific mechanism of MSCs transforming into HLCs is still unclear. At present, studies suggest that it may be related to processes such as mesenchymal-to-epithelial transition [37], Wnt signal pathway [38], and DNA methylation [39]. However, the existing studies are limited to early preclinical models, which still need further large-scale experiments to confirm.

### 2.2. Regenerative Capacity of MSCs

MSCs can participate in liver regeneration through different ways. On the one hand, MSCs can be induced to differentiate into hepatocytes under certain conditions and directly help the regeneration of damaged liver. Transplanted MSCs mainly differentiate into hepatocytes around the hepatic portal vein and promote liver tissue regeneration by upregulating the reproduction of hepatocytes and liver sinusoidal endothelial cells (LSECs), reducing the lipid accumulation in hepatocytes and expressions of interleukin- (IL-) 6 and IL-10 [40]. Choi et al. [41] found that the transplantation of chorionic-plate-derived mesenchymal stem cells into the liver injury rats can upregulate the proliferation of hepatocytes by increasing the expression of phosphatase of regenerating liver-1 in vivo and in vitro, thereby promoting liver regeneration. Winkler et al. [36] transplanted human BM-MSCs into NASH mice. Consequently, numerous human HLCs proliferated after 7 days of transplantation. Ishida et al. [24] transplanted adipose-derived tissue mesenchymal stem cells (ADSCs) into the NASH mouse model. The immunohistochemical results showed that the number of hepatocytes in NASH mice increased and the liver regeneration ability was enhanced. It was thought that this might be related to the promotion of hepatocyte regeneration by ADSCs through Notch signaling. Moreover, in the histological evaluation, the number of rat hepatocytes increased 1.2 times, and LSECs increased 1.6 times after treatment with MSC-conditioned medium (MSC-CM) [24]. The proliferation of host hepatocytes indicates that the ability of liver regeneration has been enhanced after MSC transplantation. On the other hand, MSCs can synthesize various cytokines and growth factors, exert paracrine effect in the liver tissue, and stimulate hepatocyte regeneration. For example, HGF is a cytokine with pleiotropic properties that can adjust the capacity of MSCs in multiplication and migration [42]. HGF is regarded as a key growth factor in hepatocyte multiplication, and it promotes rapid hepatocyte proliferation through the Wnt/*β*-catenin signaling and HGF/c-Met pathway [43]. After the injection of MSCs into the portal vein of partially hepatectomized mice, elevated levels of HGF, EGF, and fibroblast growth factor (FGF), as well as increased bile duct and hepatocyte proliferation, were observed [44]. These studies suggested that the regeneration ability of the damaged liver after MSC transplantation is enhanced, and MSCs may participate in the regeneration and repair process of MAFLD-damaged liver cells through direct differentiation and paracrine effect.

### 2.3. Immunomodulatory Effect of MSCs

Innate and adaptive immune response exerts significant effects on the progression of MAFLD to NASH and liver fibrogenesis. MSCs can regulate the corresponding effector cells of immunity. They regulate and repair damaged liver tissue through direct intercellular interaction and paracrine release of cytokines. Thus, they have a positive effect on the treatment of MAFLD.

Innate immune regulation: increasing evidence suggests that hepatic macrophages (i.e., Kupffer cells) play an important function in the transformation of MAFLD from steatosis to steatohepatitis [45]. In patients with MAFLD, macrophage infiltration in the portal veins could be observed during the early phase of inflammation and is associated with disease progression [46]. Hepatic macrophages include two types, M1 proinflammatory macrophages and M2 anti-inflammatory macrophages. The imbalanced polarization of M1 to M2 will cause liver injury and fibrosis [47]. Studies have presented that in both in vivo and in vitro, MSCs secrete cytokines and trigger the polarization from M1 to M2 [48]. These factors have been proved to reduce hepatic injury by accelerating M2 polarization. Chai et al. [49] found that after UC-MSCs were transplanted into rats with liver fibrosis, the mobilization of M1 macrophages increased, and the degree of liver fibrosis in rats was improved. They believed that MSCs might promote the transformation of M1 macrophages into M2 macrophages. M2 macrophages secreted the anti-inflammatory cytokine IL-10 and increased the apoptosis of M1 macrophages. A study revealed that [50], after transplantation of human amniotic mesenchymal stem cells into rats with liver fibrosis, the number of hepatic stellate cells (HSCs) and the number of CD68+ Kupffer cells decreased, significantly reducing the area of fibrosis in the liver. Furthermore, ADSCs can reduce the proliferation of HSCs by downregulating IL-17 expression and further inhibiting the progression of hepatic fibrosis in NASH mice [16]. Based on the above studies, we speculate that MSCs may affect the progression of MAFLD-related hepatocyte injury and fibrosis by regulating the activity of liver macrophages, and more animal model studies and clinical studies are needed to confirm this view in the future.

Adaptive immune regulation: T cell-mediated cellular immune response participate in the progression of NASH, mainly CD4+ T cells and CD8+ T cells [51]. MSCs interact directly with T cells or indirectly inhibit T cell proliferation by secreting soluble factors (such as TGF-*β*1 and prostaglandin E2) and thus mediate liver immunity [52]. MSCs are also known to have positive effects on the immune balance of liver tissue by increasing the growth of regulatory T cells (Tregs) [53]. Studies have suggested that MSCs could significantly reduce the infiltration and activation of CD4+ T cells and the number of Th1 cells in the liver and induce the proliferation and activation of Tregs in the liver to alleviate liver injury [54]. Wang et al. [17] selected CD4+ T cells to explore the immunoregulatory effect of MSCs on NASH and found that MSCs alleviated hepatic steatosis, inflammation, and hepatic fibrogenesis by inhibiting the multiplication and activation of CD4+IFN-*γ*+ T cells and CD4+IL-6+ T cells in the spleen of NASH mice; however, it did not influence CD4+IL-17+ T cell activity. A study found that after MSC transplantation, liver CD8+/CD4+ cells diminished, and the ability to secrete albumin was restored in the NASH mouse model [55]. MSCs can also reverse the fibrosis process and lobular inflammatory infiltration in MAFLD mice induced by high-fat diet (HDF) by inhibiting CD4+ T cell proliferation [18]. B cells can mediate liver injury and fibrosis and further promote the development of NASH to fibrosis [56]. Corcione et al. [57] found that MSCs may secrete soluble factors through paracrine pathway to arrest the cell cycle of B cells in G0/G1 and inhibit the proliferation of B cells. In addition, since the proliferation and activation of B lymphocytes largely depend on T lymphocytes, MSCs may indirectly affect the function of B cells through T cells [58]. MSCs may play an anti-inflammatory role in immune diseases by increasing the number of regulatory B cells (Bregs) [59]. Whether MSCs can mediate the immune regulation of damaged hepatocytes by regulating the activity of Bregs, it still needs more studies to confirm. Compared with MSC-induced Tregs, the specific mechanism of MSCs regulating Bregs production is still unclear, which may be related to the intercellular contact and the interaction between soluble factors. It has been reported that BM-MSC transplantation can increase the infiltration of Bregs and Tregs in the liver-injured mouse model, but the absence of Bregs does not reduce the liver injury, while the absence of Tregs completely inhibits the protective ability of MSCs on damaged hepatocytes, which indicates that Bregs does not play a key role in the immune regulation of MSC-mediated damaged hepatocytes [60]. More studies may be needed in the future to uncover the mechanism of Bregs in adaptive immune regulation of the liver.

### 2.4. Antifibrotic Activities of MSCs

Liver fibrosis occurs when numerous extracellular matrix (ECM) components accumulate in the liver. Some profibrotic factors such as TGF-*β*1, platelet-derived growth factor, and IL-1 encourage the activation of HSCs and production of collagen in the damaged liver, resulting in ECM deposition [61, 62]. The antifibrotic effects of MSCs on HSCs can be categorized as direct or indirect. Direct antifibrotic effects on HSCs are caused by the direct inhibition of their activity. In indirect action, MSCs modulated HSC activation by mediating the activity of immune cells. MSCs release soluble factors (TGF-*β*, HGF, etc.), inhibit the proliferative and activating effects of immune cells, and accelerate HSC apoptosis [63, 64]. Therefore, MSCs can regulate immune cells, thereby preventing fibrosis development. TGF-*β*1 is considered the major mediator of liver fibrogenesis, and ECM accumulation and progression of liver fibrogenesis are associated with TGF-*β*/Smad signaling [65]. An in vitro study found that BM-MSCs may inhibit the proliferation of HSCs and promote its apoptosis by inhibiting TGF-*β*/Smad pathway, thus reducing the degree of liver fibrosis [66]. Similarly, an in vivo study found that MSC transplantation reduced the levels of TGF-*β* released by Kupffer cells and M2 macrophages in a rat model of hepatic fibrosis and decreased the expression of *α*-smooth muscle actin (*α*-SMA) and the number of collagen fibers in hepatocytes [67]. MSCs can inhibit inflammatory cytokines and fibrosis marker genes (IL-1, TNF-*α*, etc.) in NASH mice and alleviate liver fibrosis [19]. Muto et al. [20] treated NASH mice with conditioned medium from stem cells derived from human-exfoliated deciduous teeth (SHED-CM) and found reduced TGF-*β* expression. Moreover, SHED-CM could protect the tight connection of intestinal epithelial cells, maintain the intestinal barrier function, and prevent lipopolysaccharides (LPSs) produced by intestinal microorganisms from coming into contact with the liver through the portal venous system to reduce the possibility of liver fibrosis. At the same time, MSCs also secrete TGF-*β*. On the one hand, TGF-*β* can promote the apoptosis of HSCs and alleviate the degree of liver fibrosis by inhibiting the proliferation and activation of immune cells. On the other hand, it can also promote liver fibrosis. The specific effect is still unknown, and further research is needed. But in general, the profibrosis effect of TGF-*β* is far less than the inhibitory effect of MSCs, which may alleviate the progression of liver fibrosis by inhibiting the level of TGF-*β*. MSCs can also secrete anti-inflammatory cytokines, such as IL-4 and IL-10, to regulate inflammatory response and reduce liver fibrosis. In the mouse model of liver fibrosis induced by CCL4, BM-MSCs were injected into the injured liver of mice, which significantly reduced the level of IL-17 and increased the level of IL-10, and alleviated the degree of liver fibrosis [68]. In liver fibrogenesis, the tissue inhibitor of metalloproteinase (TIMP) is expressed by activated HSCs, a specific inhibitor of MMPs, whereas MSCs could reduce liver fibrosis by upregulating MMPs, such as MMP-2, MMP-9, and MMP-13 [69], or by downregulating the expression of TIMP [70]. In addition, MSC cocultured with HSCs can decrease the reproduction of HSCs and the level of *α*-SMA through intercellular contact. This is mediated by triggering Notch signaling [71]. Based on the above study, Yano et al. [72] transplanted ADSCs into NASH-related cirrhotic mice and observed decreased infiltration of inflammatory cytokines (CD11b+, Gr-1+, etc.), improved liver fibrogenesis, and repaired damaged liver tissue. Zhou et al. [73] transplanted UC-MSCs into mice with liver fibrosis and inhibited the activation of HSCs by upregulating the expression of miR-148a-5p and inhibiting Notch signaling pathway, which alleviated liver fibrosis. This provides a meaningful idea for antifibrotic therapy of MSCs.

### 2.5. Regulation of Lipid Metabolism by MSCs

The dysregulation of lipid metabolism in hepatocytes induces lipotoxicity and promotes the development of MAFLD. Lipotoxicity also causes organelle dysfunction, such as endoplasmic reticulum stress, and mitochondrial dysfunction, and impaired autophagy finally leading to hepatic cell damage and apoptosis [74]. MSCs can improve the abnormal lipid metabolism of MAFLD by restoring endoplasmic reticulum stress and mitochondrial function. MSCs significantly improved endoplasmic reticulum stress and intracellular calcium homeostasis by upregulating sarcoplasmic reticulum Ca(2+) ATPase, inhibited the expression of lipid and cholesterol synthesis genes *Srebp1* and *Srebp2*, and effectively attenuated lipotoxicity and metabolic disorders in hepatocytes [21]. MSCs can also alleviate hepatic steatosis by improving mitochondrial function. In vivo and in vitro studies have revealed that mitochondrial transfer and activity enhancement after MSC transplantation, mitochondrial oxidative phosphorylation, and ATP levels were increased after the mitochondria enter the hepatocytes from MSCs, and the levels of reactive oxygen species (ROS), TG, and TC were reduced, which further alleviated hepatocytes steatosis [22]. Hsu et al. [75] found that the reduction of hepatic lipid deposition was associated with mitochondrial transfer to the liver by MSCs through tunneling nanotubes, which provided oxidative capacity for lipid decomposition, and MSC treatment decreased the expressions of CYP2E1 and 4-HNE in NASH mice, reduced ROS production, and decreased the level of lipid peroxidation. Furthermore, MSCs may improve hepatic lipid accumulation by reducing oxidative stress. The antioxidant SOD2-modified MSCs were delivered through the abdominal cavity to HFD-induced MAFLD mice, the liver fat content decreased significantly, and the level of Ucp1 in white adipose tissue (WAT) was upregulated, which promoted the browning of WAT. A study confirmed that MSCs improved hepatic lipid deposition by reducing oxidative stress [23]. MSCs can also improve serum lipid profiles and regulate lipid metabolism disorders by upregulating the expression of AMP-activated protein kinase (AMPK) and inducing the browning of WAT [13].

## 3. Signaling Pathways of MSCs Affecting MAFLD Progression

### 3.1. Notch Signaling Pathway

Evidence reveals that the Notch signaling pathway is valuable in the development of MAFLD. For example, the Notch signal increases the synthesis of hepatocyte fatty acids and lipid deposition by activating the mechanistic target of rapamycin complex 1 pathway [76]. The Notch signal can regulate cell proliferation and inhibit apoptosis [77]. Interestingly, MSCs have a positive effect on MAFLD through the Notch pathway. Ishida et al. [24] found that the Notch pathway decreased in hepatocytes of NASH mice, and the expression of Notch receptors 1 and 2 and HES1 is decreased. Flow cytometry analysis revealed that ADSCs significantly expressed Notch ligands JAG1, DLL1, and DLL4. The Notch signal mediated the activity of hepatocytes in NASH mice treated with MSCs, hepatocyte apoptosis was reduced, and the liver tissue was repaired and regenerated, but hepatocyte steatosis did not deteriorate. Meanwhile, the knockout of Notch ligand JAG1 by siRNA can weaken the antiapoptotic properties of ADSCs cocultured in vitro. ADSCs could regulate liver cell proliferation and reduce apoptosis in NASH mice through the Notch signaling pathway.

### 3.2. TLR4/NF-*κ*B Signaling Pathway

Numerous studies have recently found that TLR4/NF-*κ*B signaling is closely related to inflammatory responses, and NF-*κ*B participates in the pathophysiological changes of MAFLD by regulating lipid metabolism, lipid peroxidation, and immune response [78, 79]. MSCs can reduce the activation of HSCs and diminish hepatic fibrogenesis by regulating the TLR4/NF-*κ*B pathway [25]. Moreover, UC-MSCs combined with liraglutide improved liver lesions and decreased serum ALT, AST, and IL-6 levels in T2DM/MAFLD rats by downregulating the TLR4/NF-*κ*B signaling pathway. It restored impaired liver function and alleviated liver inflammation [80].

### 3.3. HNF4*α*–CES2 Signaling Pathway

HNF4*α* prevents the accumulation of liver lipids by promoting TG lipolysis and fatty acid oxidation and releasing very-low-density lipoprotein. Therefore, HNF4*α* plays an inhibitory role in the progression of NAFLD to NASH [81]. It is being proved that HNF4*α* regulated CES2 expression by the transactivation of the CES2 gene promoter. UC-MSCs control fat metabolism by upregulating the levels of fatty acid oxidation genes and downregulating the levels of adipogenesis-related genes; this may be related to the upregulation of the HNF4*α*–CES2 pathway [15]. Meanwhile, CES2 upregulation also enhanced PPAR*α* activities, and fatty acid *β*-oxidation was enhanced by inducing target genes *Cpt1b* and *Angplt4*. Thus, hepatic-specific HNF4*α* or CES2 knockout mice should be developed in the future to confirm that the HNF4*α*–CES2 pathway is crucial for UC-MSC therapy in MAFLD.

### 3.4. Sirtuin 1 (SIRT1) Signaling Pathway

The SIRT1 signaling pathway is extremely associated with oxidative stress, insulin resistance, lipid metabolism, and other MAFLD mechanisms. The activation of SIRT1 by inhibiting PPAR*γ* in adipose tissue increases fat decomposition and thus inhibits fat production [82]. In the MAFLD mouse model, the upregulation of SIRT1 expression by MSC-CM improves the antioxidant capacity and mitochondrial function of the liver, reduces the level of proinflammatory cytokines, and reduces apoptosis [26]. This may be associated with the downregulation of the cyclooxygenase-2 gene [83] and various proinflammatory factors (such as intracellular adhesion molecule, macrophage colony-stimulating factor, and TGF-*β*) [84] by SIRT1.

## 4. Mechanisms of Action of MSC-Exosomes on MAFLD

**Figure 2:**
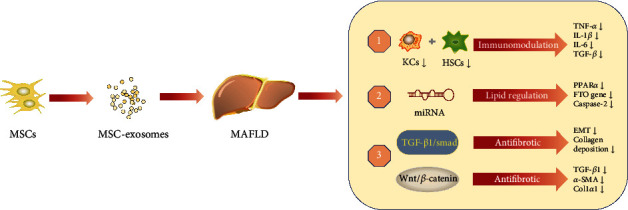
The effects of MSC-exosomes in MAFLD. MSC-exosomes play a role in MAFLD through immunomodulation, lipid regulation, and antifibrotic. MSCs: mesenchymal stem cells; MSC-exosomes: MSC-derived exosomes; KCs: Kupffer cells; HSCs: hepatic stellate cells; TNF-*α*: tumor necrosis factor-*α*; IL-1*β*: interleukin-1*β*; IL-6: interleukin-6; TGF-*β*: transforming growth factor-*β*; miRNA: microRNA; PPAR*α*: peroxisome proliferator-activated receptor-*α*; FTO gene: fat mass and obesity-related gene; *α*-SMA: *α*-smooth muscle actin; Col1*α*1: Collagen 1*α*1.

MSCs can release numerous soluble factors, and exosomes have become a research focus recently. Exosomes are extracellular vesicles with nanobilayer membranes that can be secreted by almost all cells [85]. In addition to having functions similar to MSCs, MSC-exosomes have the benefits of being a cargo delivery and having lower immunogenicity and stability in the circulation. MSC-exosomes can carry various complex components, such as proteins, lipids, miRNAs, and lncRNAs. After they enter the target cells, MSC-exosomes can regulate cell function and signal transduction [86]. MSC-exosomes also play a key role in MAFLD (Figure 2).

### 4.1. MSC-Exosomes and Immunomodulation

Recent studies have found that macrophages are primarily responsible for the immunomodulatory effect of MSC-exosomes on MAFLD. Studies have confirmed that MSC-exosomes maintain liver immune homeostasis by inducing macrophages. The active STAT3 carried by ADSC-exosomes induces high levels of expression in M2 anti-inflammatory macrophages and IL-10 by counter-activating argininase-1 and inhibited macrophage inflammatory response initiated by stimulation of LPS and interferon-*γ* [87]. More importantly, MSC-derived extracellular vesicles cannot only induce a high expression of M2 macrophages but also decrease the level of serum ALT and reverse the liver inflammation of NASH mice [88]. LPS is considered a major contributor in the worsening inflammation process in NASH [89], and LPS directly activates Kupffer cells through the TLR4 signaling pathway [90]. Amnion-derived MSC-derived extracellular vesicles (AMSC-EVs) can significantly diminish the number of Kupffer cells and downregulate the expressions of TNF-*α*, IL-1, IL-6, and TGF-*β* in the liver of NASH rats. Furthermore, AMSC-EVs significantly reduce the activation of HSCs and Kupffer cells through the LPS/TLR4 pathway and improve the degree of hepatocyte inflammation and fibrogenesis in NASH rats [91]. Ohara et al. [91] believed that AMSC-EVs can inhibit p65 and I*κ*B-*α* phosphorylation and the activities of transcription in NF-*κ*B. However, AMSC-EVs could not inhibit the transcription activities in TNF receptor-associated factor 6 from HEK293 cells, further indicating that AMSC-EVs may improve the progression of NASH through the LPS/TLR4 pathway.

### 4.2. MSC-Exosomes and Lipid Metabolism

Abnormal lipid metabolism is the main pathophysiological process that causes MAFLD [92]. Increasing evidence revealed that MSC-exosomes can affect MAFLD development through the regulation of lipid metabolism. UC-MSC-exosomes inhibited PPAR*α* and fat mass and obesity-related gene by upregulating miR-625-5p expression. It also promoted the levels of glucose-6-phosphatase, phosphoenolpyruvate carboxykinase, fatty acid synthase, and sterol regulatory element-binding transcription factor 1c (SREBP-1c), improved the glucose and lipid metabolism, reduced lipid deposition, and alleviated liver injury in MAFLD rats [93]. Other studies have revealed that UC-MSC-exosomes also improve liver lipid metabolism through autophagy. UC-MSC-exosomes activated the AMPK signaling pathway and induced elevated levels of autophagy, and the number of autophagy marker protein BECN1 and microtubule-associated protein 1 light chain 3*β* also increased, which substantially diminished the expressions of TC and TG [94]. BM-MSC-exosomes enhanced the expressions of mitochondrial autophagy-related genes *ATG5*, *ATG7*, *ATG12*, *Parkin*, *PINK1*, *ULK1*, and *BNIP3L* by upregulating miRNA-96-5p, resulting in the downregulation of its downstream target protein caspase-2 and improving hepatic steatosis [95]. In addition, M2 macrophages that have been activated by ADSC exosomes also demonstrate high levels of tyrosine hydroxylase, maintain the metabolic homeostasis of WAT, and reduce hepatic steatosis by promoting adipocyte proliferation and energy consumption [87].

### 4.3. MSC-Exosomes and Antifibrotic Effects

MSC-exosomes can reduce the development of liver fibrogenesis and induce the reversal of fibrosis. Li et al. [96] believed that MSC-exosomes restored liver fibrosis progression by downregulating the TGF-*β*/Smad pathway and inhibiting epithelial–mesenchymal transition (EMT). UC-MSC-exosomes decreased the expression of TGF-*β*1, which resulted in the phosphorylation inactivation of Smad2, reversed hepatic EMT, and reduced hepatic fibrotic collagen deposition. Human liver stem cell-derived extracellular vesicles (HLSC-EVs) can downregulate profibrotic/proinflammatory genes in NASH mice. Interestingly, among the 29 miRNAs upregulated in NASH mice, 28 returned to normal after HLSC-EV treatment, including *TGF-β1*, *α-SMA*, and collagen 1*α*1 (*Col1α1*), and these genes are related to liver fibrogenesis. Furthermore, the number of inflammatory cytokines reduced (TNF-*α*, MMP13, IL-1b, etc.) [97]. MSC-exosomes can also inhibit HSC activation and levels of *α-SMA* and *Col1α1* and reverse liver fibrosis by downregulating Wnt/*β*-catenin signaling [98].

## 5. Conclusions and Further Perspectives

MSCs have differentiation and regeneration potential, immunomodulatory properties, antifibrotic function, and regulation of lipid metabolism, and it is critical to the inception and progression of MAFLD. In recent studies, MSC transplantation can effectively reverse hepatic injury through different pathways. In addition, MSC-exosomes can upregulate or downregulate corresponding signaling pathways by carrying complex biologically active substances, alleviate liver tissue damage, and reverse MAFLD progression.

Although several studies have emphasized that MSCs and their exosomes can be a promising treatment of MAFLD, some problems remain and should not be ignored. First, the exact molecular mechanism of MSC transplantation for the treatment of MAFLD has not yet been clarified. Second, current studies tend to involve multiple interrelated processes in the progression of MAFLD. In the future, the chief elements and molecular mechanisms of their actions should be further clarified. Third, existing studies have presented that MSCs can inhibit apoptosis to reduce liver cell damage, but we still need to further reveal the molecular mechanism of MSCs inhibiting apoptosis, which is beneficial to the improvement of the therapeutic effect.

To better clarify the mechanisms involved in MSC transplantation, identifying the complex molecular cargo released by MSCs and their exosomes, such as miRNAs, lncRNAs, and proteins, is extremely important. Transcriptomics and proteomics may play a key role in exploring the potential mechanisms. In conclusion, future studies should concentrate on revealing the molecular mechanisms of MSC transplantation for the treatment of MAFLD, which will help guide the clinical treatment of MAFLD.

## Figures and Tables

**Table 1:** Animal experiments of MSC transplantation in therapy of MAFLD.

MSC source	Treatment/model	Animal	Mechanisms	Main results	Ref.
Human UC-MSCs	Leptin receptor-deficient/T2DM MAFLD	Mice	Upregulation of the HNF4*α*-CES2 signaling pathway and regulate the expression of genes related to lipid metabolism	Alleviate hepatic steatosis and improve liver injury	[15]
Mice ADSCs	HFD/NASH	Mice	Inhibit the proliferation of HSCs and decrease the level of IL-17 and the expressions of TGF-*β*1, TGF-*β*2, and *α*-SMA	Inhibit liver fibrosis	[16]
Mice BM-MSCs	MCD/NASH	Mice	Inhibit CD4+IFN-*γ*+ and CD4+IL6+ T cell proliferation and activation	Inhibit liver fibrosis and liver inflammation	[17]
Mice BM-MSCs	HFD/MAFLD	Mice	Inhibit CD4+ T cell proliferation	Inhibit liver fibrosis and liver inflammation	[18]
Mice BM-MSCs	HFD/NASH	Mice	Inhibit the expressions of proinflammatory cytokines and fibrosis-associated genes (IL-1*β*, TNF-*α*, and TGF-*β*1)	Inhibit liver fibrosis	[19]
Human SHED-MSCs	CCL4/NASH	Mice	Protect intestinal barrier function and inhibit TLR4 gene level through the gut-liver axis	Inhibit liver fibrosis and liver inflammation	[20]
Rat BM-MSCs	HFD/MAFLD	Rats	Upregulate SERCA2 and improve endoplasmic reticulum stress and intracellular calcium homeostasis	Decrease hepatocyte lipotoxicity and metabolic disorder	[21]
Mice BM-MSCs	HFD/T2DM MAFLD	Mice	Improve mitochondrial dysfunction and decrease the level of reactive oxygen species	Restore liver function and decrease hepatic steatosis	[22]
Human UC-MSCs	HFD/MAFLD	Mice	Decrease oxidative stress and inhibit the level of TNF-*α*	Decrease hepatic steatosis and hepatic inflammation	[23]
Mice ADSCs	HFD/NASH	Mice	Upregulation of the Notch signaling pathway and the expression of transcription factor HES1	Activate hepatocyte proliferation and inhibit apoptosis	[24]
Human UC-MSCs	HFD/T2DM MAFLD	Rats	Downregulation of the TLR4/NF-*κ*B signaling pathway and decrease the levels of ALT, AST, IL-6, and TNF-*α*	Improve glucose and lipid metabolism, insulin resistance, and liver injury	[25]
Human UC-MSCs	HFD/T2DM MAFLD	Mice	Upregulation of the SIRT1 signaling pathway and decrease the levels of COX-2, ICAM-1, CSF-1, and TGF-*β*	Improve liver antioxidant capacity, improve mitochondrial function, and reduce apoptosis	[26]

## Data Availability

Data sharing is not applicable to this article as no new data was created or analyzed in this study.
